# Healthcare utilization and 30-day in-hospital costs following brain tumor surgery: A single-center, retrospective cohort study

**DOI:** 10.1093/nop/npag053

**Published:** 2026-06-03

**Authors:** Floor R Pijl, Maik L Landers, Tanvi Kamra, Yasmine Zallali, Karan O T Ramcharan, Maaike J Vos, Thijs van der Vaart, Johan A F Koekkoek, Jaap D Zindler, Mar Rodríguez-Girondo, Rob J A Nabuurs, Marike L D Broekman, Wouter A Moojen, Wilco C Peul, Jeroen T J M van Dijck, Melissa Kerkhof, Rishi D S Nandoe Tewarie

**Affiliations:** Department of Neurosurgery, University Neurosurgical Centre Holland, Haaglanden Medical Centre, The Hague, The Netherlands; Department of Neurosurgery, University Neurosurgical Centre Holland, Haaglanden Medical Centre, The Hague, The Netherlands; Department of Neurosurgery, University Neurosurgical Centre Holland, Haaglanden Medical Centre, The Hague, The Netherlands; Department of Neurosurgery, University Neurosurgical Centre Holland, Haaglanden Medical Centre, The Hague, The Netherlands; Department of Neurosurgery, University Neurosurgical Centre Holland, Haaglanden Medical Centre, The Hague, The Netherlands; Department of Neurology, Haaglanden Medical Centre, The Hague, The Netherlands; Department of Neurology, Leiden University Medical Centre, Leiden, The Netherlands; Department of Neurology, Haaglanden Medical Centre, The Hague, The Netherlands; Department of Neurology, Leiden University Medical Centre, Leiden, The Netherlands; Department of Radiation Oncology, Haaglanden Medical Centre, The Hague, The Netherlands; Department of Medical Statistics, Leiden University Medical Centre, Leiden, The Netherlands; Department of Neurosurgery, University Neurosurgical Centre Holland, Haaglanden Medical Centre, The Hague, The Netherlands; Department of Neurosurgery, University Neurosurgical Centre Holland, Leiden University Medical Centre, Leiden, The Netherlands; Department of Neurosurgery, University Neurosurgical Centre Holland, Haaglanden Medical Centre, The Hague, The Netherlands; Department of Neurosurgery, University Neurosurgical Centre Holland, Leiden University Medical Centre, Leiden, The Netherlands; Department of Neurosurgery, University Neurosurgical Centre Holland, Haaglanden Medical Centre, The Hague, The Netherlands; Department of Neurosurgery, University Neurosurgical Centre Holland, Leiden University Medical Centre, Leiden, The Netherlands; Department of Neurosurgery, University Neurosurgical Centre Holland, Haaglanden Medical Centre, The Hague, The Netherlands; Department of Neurosurgery, University Neurosurgical Centre Holland, Leiden University Medical Centre, Leiden, The Netherlands; Department of Neurosurgery, University Neurosurgical Centre Holland, Haaglanden Medical Centre, The Hague, The Netherlands; Department of Neurosurgery, University Neurosurgical Centre Holland, Leiden University Medical Centre, Leiden, The Netherlands; Department of Neurology, Haaglanden Medical Centre, The Hague, The Netherlands; Department of Neurosurgery, University Neurosurgical Centre Holland, Haaglanden Medical Centre, The Hague, The Netherlands; Department of Neurosurgery, University Neurosurgical Centre Holland, Leiden University Medical Centre, Leiden, The Netherlands

**Keywords:** brain tumor surgery, cost drivers, healthcare consumption, in-hospital costs

## Abstract

**Background:**

Brain tumors cause more than 248 000 deaths annually, and their incidence continues to rise. Despite medical advances, outcomes have improved marginally, and the associated economic burden is substantial. European spending was estimated at €37.8 billion in 2019. Although several cost drivers have been identified, robust and methodologically sound cost evaluations remain limited. This study aimed to quantify 30-day in-hospital healthcare consumption and identify cost drivers in brain tumor surgery.

**Methods:**

Adult patients undergoing brain tumor surgery in 2023 at a single regional referral center were retrospectively analyzed. The primary outcome was total 30-day in-hospital costs, calculated using a bottom-up microcosting approach. Univariable and multivariable analyses were performed to identify key cost drivers.

**Results:**

A total of 322 patients (mean age 61 years; 51.2% female) were included in this study. Median length of stay (LOS) was 3 days (interquartile range; 2-6), and mean in-hospital costs were € 6988 per patient (SD ± € 4273). Tumor pathology was associated with significantly increased surgical costs (*P* = .035) but did not significantly influence overall costs (*P* = .376). Multivariable analysis identified surgery type, preoperative Karnofsky Performance Status, total LOS, intensive care unit LOS, and Clavien-Dindo complication grade as independent cost drivers. Repeat surgery, readmission, and 30-day mortality further increased costs.

**Conclusions:**

Thirty-day in-hospital costs following brain tumor surgery are substantial, mainly driven by operative and hospitalization factors. Identifying modifiable cost determinants is essential for developing targeted interventions to improve efficiency and optimize resource utilization.

Key Points

- Thirty-day in-hospital costs after brain tumor surgery were substantial, with a mean cost of € 6988 per patient.
- Costs were independently driven by surgery type, preoperative Karnofsky Performance Status, length of stay, Clavien-Dindo complication grade and 30-day outcomes.

Importance of the Study

Healthcare costs associated with brain tumor surgery have proven to be substantial. However, high-quality economic data remain scarce. This study addresses an important knowledge gap by providing a real-world retrospective analysis of 30-day in-hospital healthcare consumption using a bottom-up microcosting approach. By focusing on the perioperative period, when resource use increases, the study offers clinically relevant insights into where and why costs accrue. It demonstrates that overall costs are primarily driven by length of stay, intensive care unit (ICU) utilization, surgery type, functional status (Karnofsky Performance Scale), complication grade, and 30-day outcomes. These findings can potentially support the development of targeted strategies, including selective ICU admission, enhanced recovery pathways, and complication prevention. As healthcare systems face increasing financial pressure, this work provides an evidence base to guide efficient resource allocation while maintaining high-quality patient care.

Brain tumors are a leading cause of death worldwide, accounting for approximately 248 500 deaths each year.^1^ Incidence rates have increased from 3.75 to 4.28 per 100 000 persons over the past 2 decades, reflecting both an aging population and improved diagnostic sensitivity.^2^ The 5-year survival for all brain tumors averages at 76.5%, however remains poor for malignant types (36%)^3^ and particularly for glioblastoma (7%),^3^^,^^4^ despite several neuro-oncological developments.

Over the past decade, rapid technological and therapeutic advances have improved diagnostic accuracy and treatment possibilities.^5^^,^^6^ However, these innovations have not consistently translated into improved long-term outcomes, and remain associated with substantial costs.^2^ In Europe, brain tumor-related expenditures were estimated at €37.8 billion in 2019, a figure expected to increase with population aging and expanding treatment options.^7^ Data from the United States indicate a substantial increase in healthcare expenditures over recent decades, with the population aged >65 years exhibiting an annual percentage change (APC) of 8.8% between 1996 and 2013.^8^ Addressing this rising trend is crucial to ensure efficient and sustainable healthcare delivery, and to safeguard equitable global access to care.^9-13^

Inpatient care, in particular postoperative neurosurgical and intensive care admission, constitutes a major contributor to in-hospital costs.^14^ Existing literature identified the price and intensity of care as the primary contributors to this increase, with an annual percentage change of 11.8% observed across all age groups.^8^ A recent review on malignant brain tumors reported that current economic evaluations are limited by heterogeneous methodologies and incomplete clinical data.^15^ These limitations prohibit accurate cost outcome assessment and economic evaluation of brain tumor treatment, including surgical intervention.^16-18^ Consequently, they hinder the optimal allocation of healthcare resources.

Hence, the objective of this study is to accurately investigate the 30-day healthcare consumption and actual in-hospital costs in brain tumor surgery patients, and identify main cost drivers, to enable future economic healthcare optimization.^19-21^

## Methods

This cost analysis study was conducted with reference to the guidelines of the Consolidated Health Economic Evaluation Reporting Standards 2022 (CHEERS2022) Statement (Table S1) to enhance transparency and reporting quality.^22^ The responsible research ethics committee provided ethical approval (study number N24.018).

### Study Design and Patient Selection

This single-center retrospective study was conducted at Haaglanden Medical Center, a supraregional referral hospital in The Hague, The Netherlands, Europe. This hospital is a tertiary referral center, performing an average of 300 brain tumor operations each year, and 1 of 12 Dutch centers performing glioma surgery. Patients were included according to the following criteria: (1) age ≥18 years at the time of surgery and (2) brain tumor surgery between 1 January and 31 December 2023. A post-COVID-19 cohort was selected to ensure comparability with the contemporary healthcare environment. All tumor pathologies were included. Patients were identified through their corresponding diagnosis-treatment combination.

### Data Collection

Data were retrospectively obtained from the electronic health records. Automated extraction was used to collect baseline variables, including diagnosis-treatment combination, sex, date of birth, age, and date of surgery. Additional clinical and perioperative variables were manually retrieved using a predefined data extraction form. Data included: tumor pathology, body mass index, obesity, surgical characteristics (eg duration and type of surgery), preoperative Karnofsky Performance Scale (KPS), American Society of Anesthesiologists (ASA) score, healthcare consumption parameters (eg length of stay, imaging), in-hospital complications, and all-cause mortality. Follow-up was limited to 30 days after the surgery. All data were anonymized and stored in a secure Castor Database (Castor v2025.3.0.0).

### In-Hospital Costs

The primary outcome was 30-day in-hospital costs, defined as all inpatient and outpatient expenses incurred in the 30 days from initial surgical intervention. Costs were calculated from a healthcare perspective in accordance with the Dutch National Healthcare Institute guidelines.^23^^,^^24^ Reference prices were obtained from these guidelines (Table S2). When specific reference prices were unavailable, average unit costs were derived from the hospitals’ financial records by the hospital’s financial controllers.

A bottom-up microcosting approach was applied to quantify healthcare resource consumption.^23^^,^^25^ Total costs were calculated by unit by its respective unit cost. Healthcare use was categorized into the following components: surgery, hospital admission, imaging (CT, MRI, X-ray, and ultrasound), allied health services (physiotherapy, occupational therapy, speech therapy, and dieticians), consultations, outpatient visits, and emergency room visits. This approach provides cost estimates based on real-time resource use, rather than predetermined fees or standardized billing rates.^25^ Laboratory testing and medication costs were not included for their supposed limited contribution to total costs and obvious pragmatic reasons. All costs were expressed in Euros (2022). No discount rates were applied. No modeling was used.

### Statistical Analysis

Patient characteristics were summarized using descriptive statistics. Categorical variables were presented as counts and percentages, and continuous variables as mean ± standard deviation (SD), or median with interquartile range (IQR), as appropriate. Cost and length of stay distributions were evaluated using means and histograms.

Univariate analysis included demographic and clinical factors; tumor type (meningioma, glioblastoma, metastasis), sex, age, obesity, surgery type, ASA-score, preoperative KPS, total and ICU LOS, 30-day repeat surgery, readmission, mortality, complications, and Clavien-Dindo (CD) grade. Analyses were performed using a generalized linear model (GLM) with a Gamma distribution and log-link, chosen for its suitability for right-skewed data.^26^ Sensitivity analysis compared results to a Tweedie model. Model fit was assessed using the Bayesian information criterion and Akaike’s information criterion, with the Gamma distribution providing optimal fit. When patients experienced multiple complications across the CD subgroups, they were classified according to the most severe complication, as this was considered the most likely to result in the greatest increase in costs. Due to the limited amount of missing data, a complete case analysis was conducted.

Multivariable analysis was performed using the same Gamma distribution and log-link-based GLM. Variables significant in univariate analysis were entered in adjusted GLMs, controlling for clinically relevant confounders (ie those expected to influence outcomes based on clinical associations) (Table S4). Multivariable analyses were performed twice: once including LOS and/or ICU LOS as clinically relevant confounders, and once excluding these variables. This approach allowed explicit quantification of the extent to which cost variation was driven by hospital stay-related components. Collinearity was evaluated using variance inflation factors, all <3.

Results were reported as regression coefficient (*β*), standard error (SE), 95% confidence interval (CI), and *P-*value (*P* < .05 was considered significant for all performed ­analyses). Analyses were performed using IBM SPSS Statistics 29.0, and graphs were generated using GraphPad Prism 10.6.1.

## Results

### Patient Characteristics

A total of 322 patients were included, of which 51.2% were female (Table 1). The mean age was 61.0 years (SD 13). The most common tumor pathologies were metastasis (32.9%), glioblastoma (32.6%), and meningioma (12.1%). Most patients underwent a primary resection (80.8%), followed by a biopsy (16.1%) and repeat resection (3.1%). The majority of patients were classified as ASA-II-III (94.4%), and preoperative KPS>70 was seen in most patients (KPS 70: 14.6%; KPS 80: 30.6%; KPS 90: 29.8%; KPS 100: 9.9%).

**Table 1.** Patient characteristics.

Characteristic	*n* (%)
Sex	
Female	165 (51.2)
Male	157 (48.8)
Age	61.03 years + 13
BMI	26.15 + 5.09
Obesity (BMI > 30)	71 (22.1)
Missing	1 (0.3)
Tumor pathology	
Metastasis	106 (32.9)
Glioblastoma	105 (32.6)
Meningioma	39 (12.1)
Lymphoma	18 (5.6)
Oligodendroglioma	13 (4.0)
Astrocytoma	12 (3.7)
Glioma (NOS)	6 (1.9)
Hemangioblastoma	4 (1.2)
Ependymoma	3 (0.9)
Other	16 (5.0)
Surgery	
Biopsy	52 (16.1)
Resection	260 (80.8)
Repeat resection	10 (3.1)
ASA-score	
1	9 (2.8**)**
2	172 (53.4)
3	132 (41.0)
4	8 (2.5)
Missing	1 (0.3)
Preoperative KPS	
30	1 (0.3)
40	7 (2.2)
50	13 (4)
60	28 (8.7)
70	47 (14.6)
80	98 (30.4)
90	96 (29.8)
100	32 (9.9)
30-day readmission	13 (4.0)
30-day repeat surgery	12 (3.7)
30-day mortality	12 (3.7)
30-day complications (number of patients)	79 (24.5)
Total number of complications	98
Neurological deficit	47 (59.5)
Surgical site infection	7 (8.9)
CFS leakage	6 (7.6)
Seizure	6 (7.6)
UTI	4 (5.1)
Other	18 (18.4)
Clavien-Dindo grade	
CDI	47 (48.0)
CDII	26 (26.5)
CDIII	16 (16.3)
CDIV	4 (4.1)
CDV	5 (5.1)

Categorical categories are presented as the number of patients (*n*) and the percentage of the total number of patients (%). Continuous variables are presented as mean and standard deviation (SD). Thirty-day complication demonstrates the total number of patients who experienced one or multiple complications within 30 days. The total complications and Clavien-Dindo grade illustrate the total amount of complications that were incurred within 30 days, divided into the different subgroups.

Abbreviations: ASA, American Society of Anesthesiologists; BMI, body mass index; CD grade: Clavien-Dindo grade; CFS, cerebrospinal fluid; KPS, Karnofsky Performance Scale; NOS, not otherwise specified; UTI, urinary tract infection.

Within 30 days, 13 patients (4.0%) were readmitted, and 12 (3.7%) underwent a second surgery. Postoperative complications were most commonly CD grades I-II (74.5%) and were predominantly neurological deficit (59.5%) or surgical site infection (8.9%). Complications requiring surgical or radiological intervention (CD ≥ 3) occurred in 16.3% of patients. Finally, the 30-day mortality rate was 3.7% (*n* = 12). Missing data were limited, with both BMI and ASA-score data being unaccounted for once.

### Thirty-day In-Hospital Costs and LOS

**Figure 1.**
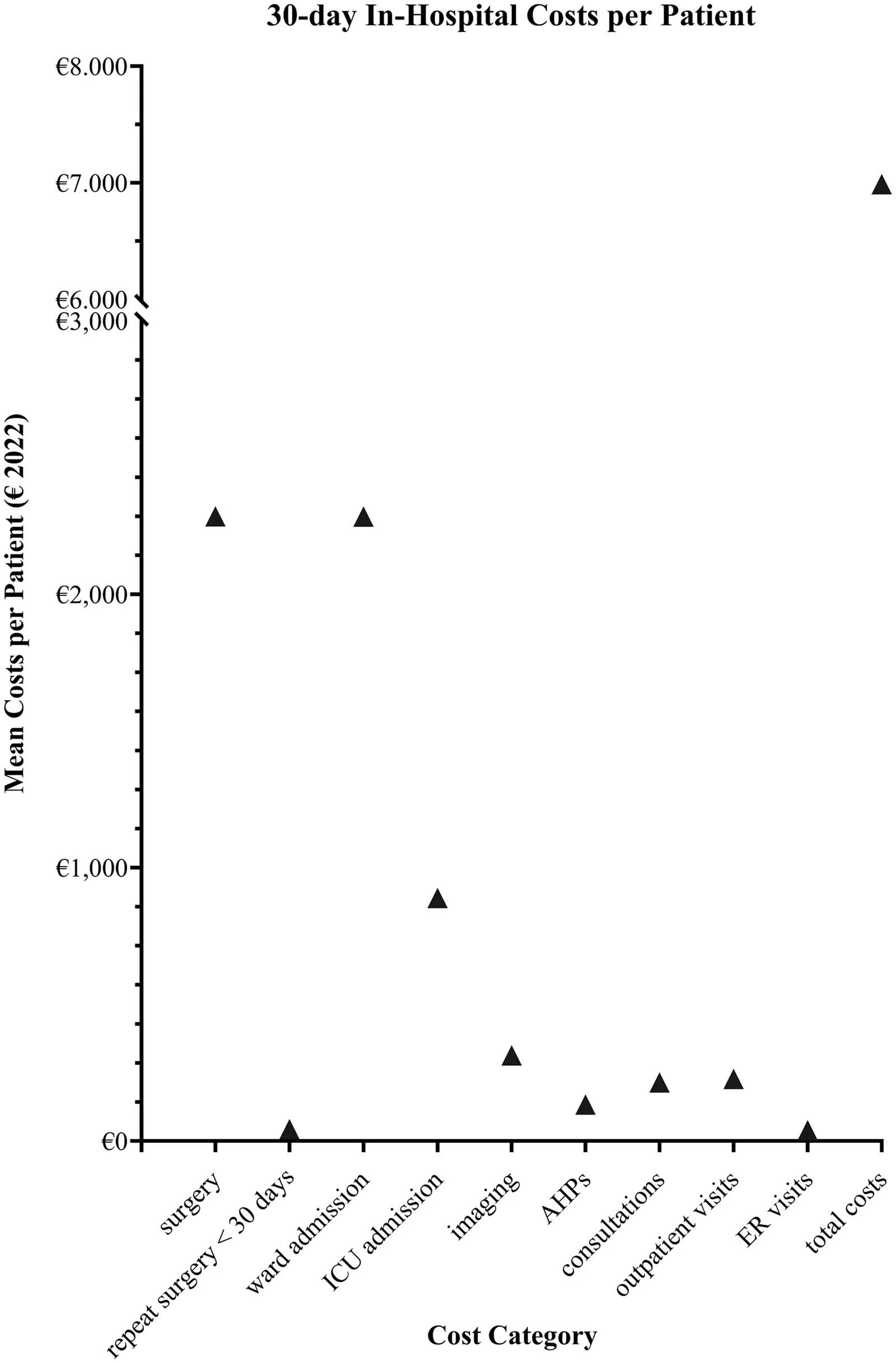
Thirty-day in-hospital costs per cost category. The figure illustrates 30-day in-hospital costs per patient for each cost category. Costs were calculated in 2022 Euro’s (€). Abbreviations: AHPs, allied health professionals; ER, emergency room; ICU, intensive care unit.

The total 30-day in-hospital cost in our cohort was € 2 250 135, corresponding to a mean of € 6988 per patient (SD ± € 4273) (Table 2, Figure 1). The main cost components were surgical procedures (€ 2285) and ward admission (€ 2284). Intensive care unit admission also contributed substantially, with a total of € 286 335, despite low patient numbers (*n* = 92). Total incurred expenditures were also influenced by imaging (€ 313 per patient).

**Table 2.** Thirty-day in-hospital costs.

	Mean 30-day in-hospital costs per patient (±SD)	Cumulative 30-day in-hospital costs (% of total)
Category		
Surgery	€ 2285 (± € 957)	€ 735 910 (32.7)
Ward admission	€ 2284 (± € 2479)	€ 917 056 (40.8)
ICU admission	€ 889 (± € 1672)	€ 286 335 (12.7)
Imaging	€ 313 (± € 271)	€ 100 684 (4.47)
Allied health professionals	€ 133 (± € 149)	€ 42 766 (1.90)
Consultations	€ 214 (± € 812)	€ 68 859 (3.06)
Outpatient visits	€ 227 (± € 232)	€ 72 960 (3.24)
ER visits	€ 39 (± € 154)	€ 11 868 (0.53)
Repeat surgery <30 days	€ 43 (± € 272)	€ 13 696 (0.61)
Total 30-day in-hospital costs	€ 6988 (± € 4273)	€ 2 250 135

Total costs and costs per patient were calculated for each category. Imaging costs consist of cumulative costs incurred with MRI scans, CT scans, X-rays, and ultrasounds. Costs per patient are displayed as means with standard deviation (SD). Costs were calculated in 2022 Euros (€).

Abbreviations: ER, emergency room; ICU, intensive care unit; LOS, length of stay; SD, standard deviation.

Total median LOS was 3 days (IQR; 2-6). When patients were admitted to the ICU, the median LOS was 1 (IQR; 1-1). Seventy-one percent of patients were not admitted to the ICU (Table 2). The average duration of all surgical intervention types is displayed in Table S5.

### Tumor Pathology

The most common diagnoses were metastasis (*n* = 106), glioblastoma (*n* = 105), and meningioma (*n* = 39), accounting for 77.6% of all cases (Table 1). Surgical costs were significantly higher in meningioma patients, with a median of € 2271 (*P* = .042). Median imaging costs were substantially lower in the meningioma cohort, due to the majority of patients not requiring postoperative imaging. However, no statistically significant difference was detected in the total imaging costs per pathology (*P* = .410). Median ICU costs for the entire cohort were substantially lower than median ward admission costs, reflecting that most patients were not admitted to the ICU in all subgroups. Intensive care unit expenditures were significantly lower in the glioblastoma group compared to both the meningioma and metastasis subgroups (*P* = .018) (Table 3, Figure S1). Median LOS did not differ significantly between the groups (*P* = 0. 244), with the longest median LOS observed in glioblastoma patients (4 days [IQR; 2-6)] (Table 3).

**Table 3.** Comparative costs and LOS per patient per tumor pathology.

	Meningioma (*n*=39)	Glioblastoma (*n*=105)	Metastasis (*n*=106)	*P*-value
Surgery costs	€ 2771 (± € 1041)	€ 2352 (± € 994)	€ 2320 (± € 662)	.042^a^
Total admission costs	€ 3513 (± € 3558)	€ 3608 (± € 2889)	€ 4062 (± € 3231)	.347
Ward costs	€ 2394 (± € 2105)	€ 3140 (± € 2646)	€ 2801 (± € 2429)	.084
ICU costs	€ 1119 (± € 2778)	€ 468 (± € 1033)	€ 1261 (± € 1732)	.018^a^
Imaging costs	€ 66 (± € 174)	€ 352 (± € 250)	€ 413 (± € 255)	.410
Total 30-day	€ 6831 (± € 4113)	€ 7067 (± € 4119)	€ 7478 (± € 4221)	.535
In-hospital costs				
Total LOS	3 (3-4)	4 (2-6)	3 (3-6)	.244

Costs per patient were calculated as means with standard deviations (SD). LOS was calculated as median with interquartile range (IQR). Costs were calculated in 2022 Euros (€). LOS was calculated in days. A generalized linear model (GLM) with a Gamma distribution and log-link were used to evaluate statistically significant differences. *P*<.05 was deemed statistically significant.

Abbreviation: LOS, length of stay.^a^The statistically significant *P*-values are marked with “a”.

### Cost Drivers

Univariate analysis identified several variables significantly associated with total 30-day in-hospital costs (Table 4). After multivariable adjustment, 8 independent cost drivers remained significant: surgery type, preoperative KPS, total LOS, ICU admission, CD grade, 30-day readmission, 30-day reoperation, and 30-day mortality (Table S3).

**Table 4.** Univariate analysis.

Characteristic	*n* (%)	30-day in-hospital costs (SD)	*P*-value
Sex			
Female	165 (51.2)	€ 7118 (± € 4406)	
Male	157 (48.8)	€ 6851 (± € 4139)	.526
Age			
18-39	23 (7.1)	€ 6987 (± € 3489)	
40-59	101 (31.4)	€ 7054 (± € 3846)	
60-79	183 (56.8)	€ 7013 (± € 4653)	
80+	15 (4.7)	€ 6239 (± € 3442)	.873
Obesity			
Yes	71 (22.1)	€ 6792 (± € 4397)	
No	250 (77.6)	€ 7043 (± € 4254)	.618
Surgery type			
Biopsy	52 (16.1)	€ 3705 (± € 2921)	
Resection	258 (80.1)	€ 7601 (± € 4219)	
Repeat resection	10 (3.1)	€ 8142 (± € 4454)	**<**.001^a^
ASA-score			
1-2	181 (56.4)	€ 6636 (± € 4011)	
3-4	140 (43.6)	€ 7441 (± € 4580)	.059
KPS, preoperative			
<70	49 (15.0)	€ 7836 (± € 4758)	
70-80	145 (45.2)	€ 7566 (± € 4846)	
>80	128 (39.9)	€ 6009 (± € 3058)	**<**.001^a^
LOS	—	—	**<**.001^a^
ICU LOS	—	—	**<**.001^a^
30-day repeat surgery			
Yes	12 (3.7)	€ 17 450 (± € 5369)	
No	310 (96.3)	€ 6583 (± € 3678)	**<**0.001^a^
30-day readmission			
No	309 (96.0)	€ 6723 (± € 3837)	
Yes	13 (4.0)	€ 13 299 (± € 8106)	**<**0.001^a^
30-day mortality			
No	310 (96.3)	€ 6762 (± € 3978)	
Yes	12 (3.7)	€ 12 814 (± € 7066)	**<**0.001^a^
Complications			
No	243 (75.5)	€ 5927 (± € 2827)	
Yes, one	67 (20.8)	€ 9404 (± € 5418)	
Yes, multiple	12 (3.7)	€ 14 979 (± € 7136)	**<**0.001^a^
Clavien-Dindo grade			
CD0	243 (75.5)	€ 5927 (± € 2827)	
CDI-II	59 (18.3)	€ 8692 (± € 4837)	
≥CDIII	20 (6.2)	€ 14 850 (± € 6846)	**<**0.001^a^

Costs were calculated as means with standard deviations (SD) using descriptive statistics. Costs were calculated in 2022 Euro’s (€). *P-*value were calculated utilizing a generalized linear model (GLM) with a Gamma distribution and log-link. *P*<.05 was deemed statistically significant.

Abbreviations: ASA, American Society of Anesthesiologists; CD grade, Clavien-Dindo grade; ICU, intensive care unit; KPS, Karnofsky Performance Scale; LOS, length of stay; SD, standard deviation.^a^The statistically significant *P*-values are marked with “a”.

The highest costs were observed in patients who underwent a reoperation, with unadjusted and adjusted mean costs of € 17 450 and € 13 795, respectively (Table 4, Table S3). Intensive care unit length of stay was the strongest predictor of cost, with each additional ICU-day associated with a 39.6% increase in total costs (*β* = 0.396), compared to a 10% increase per additional hospital day (*β* = 0.100) (Table S3).

A preoperative KPS < 70 was associated with a 36.6% increase in total costs. The type of surgery also strongly influenced costs, with resection leading to 62.8% higher costs than biopsy (€ 7601 vs € 3705). Clavien-Dindo grade III complications were associated with nearly double the total cost (€ 14 979 vs € 5927 for CD0).

Patients who died within 30 days had higher total costs (€ 12 814 vs € 6762), likely caused by extended hospital stay (median 3 days vs 7.5 days). In addition, readmission and repeat surgery showed a significant correlation to total 30-day in-hospital costs (Table S3).

## Discussion

This study provides a systematic evaluation of 30-day in-hospital costs associated with brain tumor surgery, identifying LOS, ICU LOS, surgical procedure, preoperative KPS, and CD grade as significant cost drivers after adjustment for confounding variables. In addition, 30-day readmission, reoperation, and 30-day mortality were significantly associated with higher total 30-day in-hospital expenditures.

When compared with previously published data, our findings suggest similar mean 30-day in-hospital costs (€ 6988) relative to the lower range in prior reports, which ranged between $6206 and $30 836 in high-income countries.^16^^,^^21^^,^^27^ Higher costs compared to existing literature were initially expected due to inflation and the rise of healthcare expenses over the years, contrasting with our findings. Previous studies have demonstrated a substantial increase in healthcare costs over recent decades, driven by rising prices and the intensity of care. Literature reports an APC of +11.8% between 1996 and 2013. In addition, pharmaceutical expenditures exhibited an APC of +8.7%, largely attributable to increasing drug prices.^8^^,^^28^ These findings underscore the significant contribution of inflation and rising pricing to overall healthcare expenditure growth.

The discrepancies with our findings are likely attributable to differences in methodology, as most prior studies used aggregated top-down cost estimates and rarely disclosed cost calculation strategies. Moreover, cost-saving policies like extended stay at the postanesthesia care unit, leading to decreased ICU admission for postoperative patient monitoring, are explanations for this contradiction. Our bottom-up microcosting approach offers greater precision and transparency and is widely regarded as the gold standard in economic evaluations.^23^ Although we aimed to capture all relevant cost components, certain minor elements, such as laboratory testing and medication use, were not included.

Differences in healthcare system structure may also contribute to variation in reported 30-day in-hospital costs for brain tumor treatment. Healthcare systems vary substantially in terms of funding mechanisms, reimbursement models, and resource allocation, all of which can influence cost estimates.^29^^,^^30^ For example, systems based on diagnosis-related groups or bundled payments may incentivize shorter hospital stays and cost containment, whereas fee-for-service models may be associated with higher resource utilization and, consequently, higher costs.^29^ Differences in labor costs, infrastructure, and national pricing regulations, particularly for pharmaceuticals and medical devices, could also potentially be fundamental.^8^^,^^29^ These structural variations limit direct comparability between studies conducted in different healthcare settings and should be carefully considered when interpreting cost analyses. Currently, insufficient literature is available to evaluate these influences on the cost of brain tumor surgery.

Notably, tumor pathology did not significantly affect overall incurred costs in our cohort. Although surgical and ICU costs differed significantly, total 30-day costs did not. Even though differences were expected due to heterogeneity in disease care paths, this was not illustrated in our cost data. Meningioma patients displayed the highest surgical costs. These patients often do not require postoperative imaging, reflected by the median € 0 imaging costs, which may account for the nonsignificant difference in total costs. Current literature provides limited evidence regarding this topic. One small single-center study from Brazil found no statistically significant differences in direct neurosurgical costs between adults with meningioma and those with glioma.^31^

Length of stay was a major cost determinant. The median LOS in our study, 3.00 (IQR; 2.00-6.00), was slightly lower than earlier reports (4-6 days).^16^^,^^27^^,^^32^ These differences may reflect evolving perioperative management, minimally invasive surgical techniques, and adoption of early discharge protocols.^16^^,^^18^^,^^32-34^ Given the substantial daily tariffs associated with ICU admission,^23^ the strong link between ICU stay and cost escalation aligns with previous findings in neurosurgical populations.^35^^,^^36^ These results underscore that shortening total and ICU LOS represents one of the most impactful measures for cost reduction.

Surgical procedure type also significantly influenced costs. Resection and repeat resections incurred substantially higher expenditures than biopsies, largely due to longer operative times (Table S4) and higher complication risks.^37-39^ These differences are consistent with literature showing that tumor size, location, and pathology influence surgical complexity, duration, and costs.^37^

Preoperative KPS emerged as another independent cost driver, reflecting the importance of preoperative functional status and frailty on postoperative patient outcomes. Patients with lower KPS (<70) have higher complication rates and increased hospital LOS.^40^ Although comorbidities were not directly adjusted for in our study, preoperative KPS remains a robust and clinically meaningful predictor of healthcare utilization and costs.

Complications were strongly associated with increased in-hospital costs. Previous studies in general surgery have reported a rise in-hospital costs with increasing CD grade, particularly for CD >III complications that require reintervention.^41-43^ Current neurosurgical research displays increased costs when complications occur, but data on the effect of the CD grade is scarce.^20^^,^^21^ It does demonstrate a higher incidence of low CD grade (CDI-II) complications, correlating to our data.^36^

Our data also demonstrated the significant association of 30-day clinical outcomes with 30-day in-hospital costs, aligning with current data. Patients readmitted within 30 days can be expected to have an increased LOS, contributing to higher expenditures. These associations extend beyond LOS, reflecting the additive impact of recurrent complications, repeat interventions, and ICU admissions. Correspondingly, patients who underwent repeat surgery within 30 days can expect higher costs due to additional interventions or increased complications. Additionally, deceased patients reported higher expenditures. Direct postoperative complications can lead to quick deterioration, with hematomas reportedly accounting for one-third of 30-day mortality.^44^ As mentioned before, this can lead to increased costs due to additional interventions or even ICU admission.

Identification of these key cost drivers highlights potential interventions to decrease future healthcare expenditures. Both prehospital and in-hospital interventions can be effective in combating costs. Firstly, the necessity of routine postoperative ICU admission should be critically evaluated. Evidence supports the safety and cost-effectiveness of a “No ICU Unless” policy.^35^^,^^36^^,^^45^ Moreover, these protocols have been proven to reduce complication rates, contributing to even lower expenditures.^35^^,^^36^ Alternatively, to reduce ward LOS, outpatient craniotomies and early discharge protocols show promising cost reductions, without compromising patient safety.^33^^,^^34^^,^^46^ Although the severity of complications is not easily modifiable, their prevalence can be effectively reduced. Implementing preventative strategies can generate a substantial cost reduction, for instance, in the prevention of surgical site infections (SSIs). Due to the substantial contribution of SSIs to complication rates, these interventions could produce tremendous effects on overall incurred costs.^47^

A holistic approach to health care has the potential to address multiple cost drivers simultaneously. These protocols, for example, enhanced recovery after surgery (ERAS), adopt personalized medicine as the central cornerstone.^48^ By optimizing nutrition and overall mobility, preoperative KPS could be improved. Moreover, accurate patient counseling and pain management, but also improved physical fitness, can decrease LOS and possibly prevent ICU admission. Lastly, improving overall patient health may reduce complication risk. This represents a future challenge that must be addressed.

### Strengths and Limitations

The main strength of our study is the use of a bottom-up microcosting approach, which enables a precise estimation of real-world expenditures. Additional strengths include reference to CHEERS 2022 reporting guidelines, little missing data (*n* = 2), and the use of multivariable generalized linear modeling to adjust for confounders. Limitations are sole inclusion of in-hospital costs, retrospective estimation of preoperative KPS in some cases, and exclusion of certain cost categories (eg laboratory testing, medication use, specific operating instruments), which may lead to underestimation of total costs. Heterogeneity in standardized care, for example, pre- and postoperative imaging protocols across tumor types, may have influenced the apparent lack of cost differences by pathology. Moreover, it is important to acknowledge the low event counts for several variables, including readmission (*n* = 13), repeat surgery (*n* = 12), mortality (*n* = 12), and CD grade ≥III complications (*n* = 20). Given the limited incidence of these events, the corresponding estimates may be unstable, particularly in adjusted models. Considering their substantial impact on total costs, reflected by the marked increase in estimated marginal means in multivariable analyses (eg 30-day repeat surgery: € 8005 vs € 14 833), this may have influenced the results. Finally, as a single-center, single-year analysis, generalizability to other healthcare systems and countries is limited. Moreover, costs are substantially dependent on center-specific protocols regarding standardized patient care, ICU admission, and discharge. Therefore, results can be more difficult to extrapolate to different healthcare systems, hospitals, or countries.

### Future Research

Future research should include multicenter cohorts to improve external validity and identify cross-system variability in cost structures. By evaluating modifiable and nonmodifiable cost determinants separately, risk markers can be optimally stratified. Moreover, it is essential to look beyond the perioperative timeframe, including adjuvant therapies, such as chemotherapy and radiotherapy, which are needed to capture the full economic burden of brain tumor treatment. Furthermore, the integration of socioeconomic determinants of health, which influence both access and outcomes, is crucial for understanding broader healthcare disparities.^16^^,^^49^ Finally, prospective trials implementing ERAS or selective ICU admission protocols are essential to evaluate their cost-effectiveness and guide evidence-based resource allocation.

## Conclusion

The mean 30-day in-hospital costs of brain tumor surgery in our center were € 6988. Costs were mainly driven by LOS, ICU stay, surgical procedure, preoperative KPS, and complication severity. These findings provide a high-quality estimate of short-term hospital costs and highlight key targets for improving efficiency and sustainability in brain tumor surgery.

## Supplementary Material

npag053_Supplementary_Data

## Data Availability

The datasets generated and analyzed during the current study are available from the corresponding author on reasonable request.
