# Supplementary material for: Healthcare utilization and 30-day in-hospital costs following brain tumor surgery: A single-center, retrospective cohort study
Source: Neurooncol Pract. 2026 Jun 3;13(5):907–17. doi: 10.1093/nop/npag053 (PMC13578511; doi:10.1093/nop/npag053)
Supplement: Supplementary file 1 [file npag053_supplementary_data.zip › Supplementary materials.docx]

**Supplementary Material**

Supplementary Table 1. CHEERS 2022 Statement

|  | **Item** | **Guidance for Reporting** | **Reported in section** |
| --- | --- | --- | --- |
| **TITLE** | | |  |
| Title | 1 | Identify the study as an economic evaluation and specify the interventions being compared. | Title, page 1 |
| **ABSTRACT** | | |  |
| Abstract | 2 | Provide a structured summary that highlights context, key methods, results and alternative analyses. | Abstract, page 2 |
| **INTRODUCTION** | | |  |
| Background and objectives | 3 | Give the context for the study, the study question and its practical relevance for decision making in policy or practice. | Introduction, page 3 |
| **METHODS** | | |  |
| Health economic  analysis plan | 4 | Indicate whether a health economic analysis plan was developed and where available. | Data collection, page 4 |
| Study population | 5 | Describe characteristics of the study population (such as age range, demographics, socioeconomic, or clinical characteristics). | Data collection, page 4 |
| Setting and location | 6 | Provide relevant contextual information that may influence findings. | In-hospital costs, page 4 |
| Comparators | 7 | Describe the interventions or strategies being compared and why chosen. | Patient selection, page 4 |
| Perspective | 8 | State the perspective(s) adopted by the study and why chosen. | In-hospital costs, page 4 |
| Time horizon | 9 | State the time horizon for the study and why appropriate. | Study design & patient selection, page 4 |
| Discount rate | 10 | Report the discount rate(s) and reason chosen. | In-hospital costs, page 5 |
| Selection of outcomes | 11 | Describe what outcomes were used as the measure(s) of benefit(s) and harm(s). | In-hospital costs and data collection, page 4 |
| Measurement of outcomes | 12 | Describe how outcomes used to capture benefit(s) and harm(s) were measured. | In-hospital costs and data collection, page 4 |
| Valuation of outcomes | 13 | Describe the population and methods used to measure and value outcomes. | In-hospital costs and data collection, page 4 |
| Measurement and valuation of resources  and costs | 14 | Describe how costs were valued. | In-hospital costs, page 4 |
| Currency, price date, and conversion | 15 | Report the dates of the estimated resource quantities and unit costs, plus the currency and year of conversion. | In-hospital costs, page 5 |
| Rationale and  description of model | 16 | If modelling is used, describe in detail and why used. Report if the model is publicly available and where it can be accessed. | In-hospital costs, page 5 |
| Analytics and assumptions | 17 | Describe any methods for analyzing or statistically transforming data, any extrapolation methods, and approaches for validating any model used. | Statistical analysis, page 5 |
| Characterizing heterogeneity | 18 | Describe any methods used for estimating how the results of the study vary for sub-groups. | Statistical analysis, page 5 |
| Characterizing  distributional effects | 19 | Describe how impacts are distributed across different individuals  or adjustments made to reflect priority populations. | Statistical analysis, page 5 |
| Characterizing uncertainty | 20 | Describe methods to characterize any sources of uncertainty in the analysis. | Statistical analysis, page 5 |
| Approach to engagement with patients and others affected by the study | 21 | Describe any approaches to engage patients or service recipients, the general public, communities, or stakeholders (e.g., clinicians or payers) in the design of the study. | In-hospital costs, page 4 and statistical analysis, page 5 |
| **RESULTS** | | |  |
| Study parameters | 22 | Report all analytic inputs (e.g., values, ranges, references) including uncertainty or distributional assumptions. | Results, page 6-13 |
| Summary of main results | 23 | Report the mean values for the main categories of costs and outcomes of interest and summarize them in the most appropriate overall measure. | 30-day in-hospital costs and length of stay, page 8 |
| Effect of uncertainty | 24 | Describe how uncertainty about analytic judgments, inputs, or projections  affect findings. Report the effect of choice of discount rate and time horizon, if applicable. | Results, page 6-13 |
| Effect of engagement with patients and others affected by the study | 25 | Report on any difference patient/service recipient, general public, community, or stakeholder involvement made to the approach or findings of the study | Tumor pathology, page 9 and cost drivers, page 11 |
| **DISCUSSION** | | |  |
| Study findings, limitations, generalizability, and current knowledge | 26 | Report key findings, limitations, ethical or equity considerations not captured, and how these could impact patients, policy, or practice. | Discussion, page 14-17 |
| **OTHER RELEVANT INFORMATION** | | | |
| Source of funding | 27 | Describe how the study was funded and any role of the funder in the identification, design, conduct, and reporting of the analysis | Funding, page 18 |
| Conflicts of interest | 28 | Report authors conflicts of interest according to journal or  International Committee of Medical Journal Editors requirements. | Conflicts of interest, page 18 |

Husereau D, Drummond M, Augustovski F, de Bekker-Grob E, Briggs AH, Carswell C, Caulley L, Chaiyakunapruk N, Greenberg D, Loder E, Mauskopf J, Mullins CD, Petrou S, Pwu RF, Staniszewska S; CHEERS 2022 ISPOR Good Research Practices Task Force. Consolidated Health Economic Evaluation Reporting Standards 2022 (CHEERS 2022) Statement: Updated Reporting Guidance for Health Economic Evaluations. BMJ. 2022;376:e067975.

Supplementary Table 2. Cost Reference Data

| **Healthcare consumption category** | **Reference price data 2023 (€ 2022)** | **Source** |
| --- | --- | --- |
| Operating room per minute | € 11,09 | Dutch National Health Care Institute^21^ |
| Admission day ward | € 644,00 | Dutch National Health Care Institute^21^ |
| ICU admission day | € 2.727,00 | Dutch National Health Care Institute^21^ |
| CT scan | € 188,00 | Dutch National Health Care Institute^21^ |
| MRI scan | € 254,00 | Dutch National Health Care Institute^21^ |
| X-ray | € 82,17 | Dutch National Health Care Institute^21^ |
| Ultrasound | € 103,00 | Dutch National Health Care Institute^21^ |
| Consultation physiotherapist | € 38,89 | Dutch National Health Care Institute^21^ |
| Consultation occupational therapist | € 24,32 | Dutch National Health Care Institute^21^ |
| Consultation dietician | € 24,70 | Dutch National Health Care Institute^21^ |
| Consultation speech therapist | € 40,93 | Dutch National Health Care Institute^21^ |
| Clinical consultation medical specialty | € 213,85 | Financial records of study hospital (internal financial controllers) |
| Outpatient consult | € 120,00 | Dutch National Health Care Institute^21^ |
| ER-visit | € 258,00 | Dutch National Health Care Institute^21^ |

Supplementary Table 3. Generalized linear model of possible cost drivers

|  | **Estimate (β)** | **SE** | **95% CI**  **Lower – Upper** | **P-value** | **EMM total cost**  **(95% CI lower – upper)** |
| --- | --- | --- | --- | --- | --- |
| **Surgery type**  Biopsy  Resection  Re-resection | **  0.630  0.661 | –  0.0863  0.1722 | – / –  0.461 – 0.799  0.323 – 0.999 | **<0.001*** | € 4479 (€ 3657 – € 5486)  € 8407 (€ 7185 – € 9837)  € 8675 (€ 6264 – € 12,014) |
| **ASA-score**  1-2  3-4 | **  0.132 | –  0.0622 | – / –  0.010 – 0.254 | **0.034** | € 6339 (€ 5652 – € 7110)  € 7235 (€ 6416 – € 8157) |
| **Preoperative KPS**  >80  70-80  <70 | **  0.181  0.357 | –  0.0639  0.0878 | – / –  0.056 – 0.307  0.185 – 0.529 | **<0.001*** | € 6140 (€ 5129 – € 7349)  € 7360 (€ 6214 – € 8718)  € 8776 (€ 7079 – € 10,879) |
| **LOS** | 0.089 | 0.0052 | 0.079 – 0.099 | **<0.001*** | – |
| **ICU LOS** | 0.375 | 0.0373 | 0.302 – 0.448 | **<0.001*** | – |
| **Clavien-Dindo grade**  CD0  CDI-II  ≥CDIII | **  0.365  0.857 | –  0.0582  0.0920 | – / –  0.251 – 0.479  0.667 – 1.038 | **<0.001*** | € 6017 (€ 5152 – € 7029)  € 8670 (€ 7216 – € 10,418)  € 14,182 (€ 11,271 – € 17,845) |
| **30-day Readmission (without LOS)**  No  Yes | **  0.484 | –  0.1088 | – / –  0.271 – 0.697 | **<0.001*** | € 10,349 (€ 8628 – € 12,413)  € 16,792 (€ 12,878 – € 21,894) |
| **30-day Readmission (with LOS)**  No  Yes | **  0.158 | –  0.0655 | – / –  0.030 – 0.287 | **0.016*** | € 5683 (€ 5080 – € 6358)  € 6657 (€ 5637 – € 7863) |
| **30-day Repeat Surgery**  No  Yes | **  0.617 | –  0.1461 | – / –  0.330 – 0.903 | **<0.001*** | € 8005 (€ 6763 – € 9475)  € 14,833 (€ 11,138– € 19,754) |
| **30-day Mortality**  No  Yes | **  0.263 | –  0.1170 | – / –  0.033 – 0.492 | **0.025*** | € 10,720 (€ 8909 – € 12,900)  € 13,941 (€ 10,490 – € 18,527) |

**Supplementary Table 3. Generalized linear model of possible cost drivers for total in-hospital costs.** For each variable of interest, a separate GLM was performed with all possible confounders included in the model.

* P-value of <0.05 was deemed statistically significant.

** This category was set as reference category.

Abbreviations: ASA: American Society of Anesthesiologists; CD grade: Clavien-Dindo grade; EMM: Estimated Marginal Means; ICU: Intensive Care Unit; KPS: Karnofsky Performance Scale; LOS: Length of Stay; 95% CI: 95% confidence interval; SE: standard error.

Supplementary Table 4. Corrected confounders per factors of interest

| **Factor** | **Confounders** |
| --- | --- |
| Surgery type | Sex  Age  Tumor pathology  ASA-score  Preoperative KPS |
| ASA-score | Sex  Age  Obesity |
| Preoperative KPS | Sex  Age  Obesity  Tumor pathology |
| LOS | Sex  Age  Obesity  Tumor pathology  Surgery type  ASA-score  Preoperative KPS  30-day repeat surgery  Clavien-Dindo grade |
| ICU LOS | Sex  Age  Obesity  Tumor pathology  Surgery type  ASA-score  Preoperative KPS  30-day repeat surgery  Clavien-Dindo grade |
| Clavien-Dindo grade | Sex  Age  Obesity  Tumor pathology  Surgery type  ASA-score  Preoperative KPS |
| 30-day readmission | Sex  Age  Obesity  Tumor pathology  Surgery type  ASA-score  Preoperative KPS  30-day repeat surgery  Clavien-Dindo grade  LOS  ICU LOS |
| 30-day repeat surgery | Sex  Age  Obesity  Tumor pathology  Surgery type  ASA-score  Preoperative KPS  Clavien-Dindo grade |
| 30-day mortality | Sex  Age  Obesity  Tumor pathology  Surgery type  ASA-score  Preoperative KPS  30-day repeat surgery  Clavien-Dindo grade |

Abbreviations: KPS: Karnofsky Performance Scale; LOS: Length of Stay.

Supplementary table 5. Duration of surgical interventions

| **Surgical intervention** | **Median duration of intervention (IQR)** |
| --- | --- |
| Resection | 224 (181 – 275) |
| Repeat resection | 209 (195 – 233) |
| Biopsy | 77 (64 – 92) |

The median duration and respective interquartile range (IQR) of all interventions. The duration is displayed in minutes.

Abbreviations: IQR; interquartile range

Supplementary Figure 1. Major Cost Categories per Tumor Pathology

**Supplementary Figure 1. Major Cost Categories per Tumor Pathology.** The graph illustrates the mean 30-day In-Hospital Costs of the most common tumor pathologies in the study cohort. The major cost categories are displayed on the x-axis. Costs are demonstrated as cost per patient and were calculated in 2022 Euro’s (€).
